# Early hippocampal hyperexcitability and synaptic reorganization in mouse models of amyloidosis

**DOI:** 10.1016/j.isci.2024.110629

**Published:** 2024-08-02

**Authors:** Ajit Ray, Iulia Loghinov, Vijayalakshmi Ravindranath, Alison L. Barth

**Affiliations:** 1 Department of Biological Sciences, Carnegie Mellon University, Pittsburgh, PA 15213, USA; 2 Centre for Neuroscience, Indian Institute of Science, Bengaluru, Karnataka 560012, India; 3 Centre for Brain Research, Indian Institute of Science, Bengaluru, Karnataka 560012, India

**Keywords:** Molecular neuroscience, Cellular neuroscience

## Abstract

The limited success of plaque-reducing therapies in Alzheimer’s disease suggests that early treatment might be more effective in delaying or reversing memory impairments. Toward this end, it is important to establish the progression of synaptic and circuit changes before onset of plaques or cognitive deficits. Here, we used quantitative, fluorescence-based methods for synapse detection in CA1 pyramidal neurons to investigate the interaction between abnormal circuit activity, measured by Fos-immunoreactivity, and synapse reorganization in mouse models of amyloidosis. Using a genetically encoded, fluorescently labeled synaptic marker in juvenile mice (prior to sexual maturity), we find both synapse gain and loss depending on dendritic location. This progresses to broad synapse loss in aged mice. Elevated hippocampal activity in both CA3 and CA1 was present at weaning and preceded this reorganization. Thus, Aβ overproduction may initiate abnormal activity and subsequent input-specific synapse plasticity. These findings indicate that sustained amyloidosis drives heterogeneous and progressive circuit-wide abnormalities.

## Highlights

• Abnormal hippocampal activity is an early stage pathology of β-amyloidosis models
• Synaptic reorganization in CA1 pyramidal neurons follows altered activity
• Juvenile mice show simultaneous gain and loss of synapses based on dendritic location
• Aged mice show a broader loss of synapses across cellular compartments

## Graphical abstract

**Figure:**
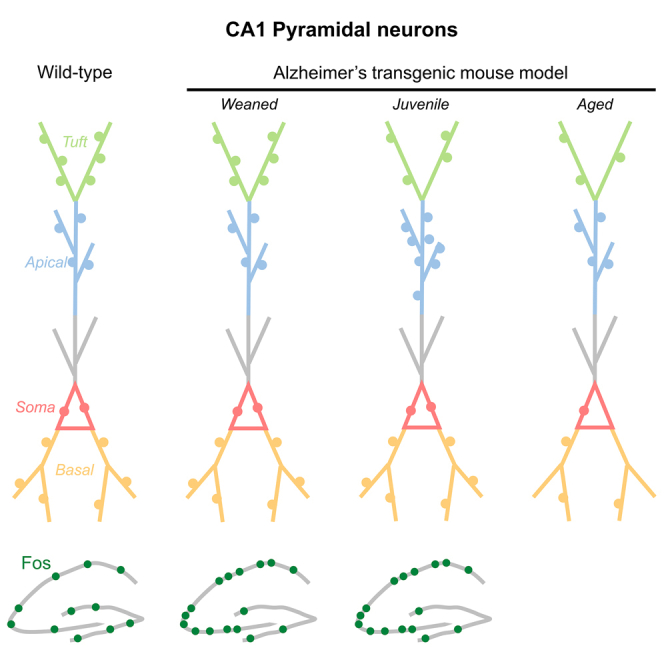

## Introduction

Alzheimer’s disease (AD) is the leading cause of dementia and is histopathologically characterized by extensive deposition of extracellular amyloid-β (Aβ) plaques and intracellular neurofibrillary tangles in the brain. Human studies suggest that Aβ aggregation starts at least 10–20 years before the onset of memory deficits and cognitive decline.^1^^,^^2^^,^^3^ Both human and animal studies indicate that neuronal hyperactivity and synaptic dysfunction in the hippocampus can precede Aβ accumulation and plaque appearance.^4^^,^^5^^,^^6^^,^^7^^,^^8^^,^^9^^,^^10^^,^^11^^,^^12^^,^^13^ Indeed, hippocampal hyperactivity is correlated with both cognitive impairment and the accumulation of amyloid plaques, further suggesting a link between these phenomena.^4^^,^^14^^,^^15^^,^^16^^,^^17^ Overall, there is increasing experimental evidence that Aβ overproduction can alter the normal function of hippocampal circuits, directly influencing both synaptic function and normal patterns of network activity. Brain pathology associated with AD may be the result of both neurodegenerative as well as neuroplastic processes, with the latter occurring at earlier stages of amyloidosis.^18^

Although AD itself is linked to profound synapse loss in affected brain regions, other synaptic abnormalities besides loss have been clinically observed in early-stage and prodromal AD.^19^^,^^20^^,^^21^ Aβ can both enhance and suppress synaptic plasticity at hippocampal synapses,^14^^,^^22^^,^^23^ and the effects of Aβ at different synapse subtypes may depend upon activity levels and molecular composition. A connection between network hyperexcitability and synaptic dysfunction has not been well-established, in part because of a bias in examination of tissue with high levels of amyloidosis where multiple cellular pathological events have already set in. Thus, it remains possible that early modifications in cellular excitability^24^ or synapse strength or number might initiate subsequent pathology.

Identification of the temporal sequence of hyperactivity and synaptic reorganization might shed light onto which factors initiate the complex cascade of events leading to cognitive impairment and disease. In this anatomical study, we sought to examine both hippocampal network activity and synaptic distribution at early developmental time-points well before plaque accumulation and significant synaptic loss as seen in older animals. By focusing on the early effects of Aβ/APP-proteolytic products overproduction in mouse models of amyloidosis, we sought to disentangle elevated network activity and synaptic alterations.

CA1 pyramidal neurons (Pyr) are ideal for examining input-specific synaptic vulnerability linked to Aβ overproduction, since presynaptic afferents are segregated according to location along the dendritic arbor.^25^ Anatomical studies in mouse models of amyloidosis indicate that synapses onto CA1 Pyr may be weakened even in young adult mice, and frank synaptic loss that accelerates with age has been observed across multiple strains.^26^^,^^27^^,^^28^^,^^29^ Typically these studies have not identified the onset of synaptic abnormalities during development, adolescence, and adulthood, and have been agnostic to potential input-specific effects across the CA1 dendritic arbor.

Structural analyses have typically employed Golgi-staining or fluorescent cell-fills to visualize dendritic spines and shown excitatory synaptic loss on CA1 Pyr during aging in a variety of amyloidosis models.^28^^,^^29^^,^^30^^,^^31^ However, such methods are unsuitable for studying inhibitory synaptic changes, as most inhibitory synapses are located on the cell soma and dendritic shaft. The development of genetically encoded fluorescent synaptic markers has facilitated quantitative synapse analysis, where reagents may label excitatory, inhibitory, or both types of synapses.^32^^,^^33^^,^^34^^,^^35^^,^^36^^,^^37^ The neuroligin-based FAPpost synaptic tag is particularly attractive as it comprehensively labels both excitatory and inhibitory postsynaptic structures in labeled neurons with a single reagent.^36^

Here, we tested whether altered hippocampal activity measured by immediate-early gene (IEG) expression precedes broader synaptic changes in adolescent hippocampal CA1 pyramidal neurons using two different mouse models of amyloidosis, APPSwe/PS1dE9 (APP/PS1) and APPSwe (Tg2576). Our results demonstrate that both CA3 and CA1 hippocampal subregions exhibited elevated activity in young animals, measured using immunoreactivity against the IEG *c-fos* (Fos-IR). These changes occur several weeks prior to changes in synapse distribution. We also report opposing changes in dendritic sub-compartments of CA1 neurons in juvenile (6-week-old) mice, with a decrease in synapses at the apical tuft, which is dominated by input from entorhinal cortex, and an increase in synapses along the apical dendrite, which receives CA3 inputs. In aged (12-15–month-old) animals, this compartment-specific reorganization of synapse density transitioned to an overall loss of synapses across all CA1 dendritic compartments.

## Results

### Juvenile APP/PS1 mice show elevated hippocampal fos expression

Abnormal activity localized to the hippocampus has been observed in individuals carrying familial AD-associated mutations decades prior to the onset of cognitive impairment,^10^ and animal models of amyloidosis also exhibit hippocampal hyperactivity that precedes plaque deposition by months.^6^^,^^9^^,^^12^ Indeed, bath application of soluble Aβ increases Ca^2+^ transients in CA1 pyramidal (Pyr) neurons, suggesting an acute effect. Aβ has also been linked to altered intrinsic excitability, both increasing and decreasing neuronal firing depending on Aβ concentration and oligomeric composition.^6^^,^^14^^,^^24^^,^^38^^,^^39^^,^^40^ These data are consistent with a model by which sustained exposure to Aβ might drive progressive alterations in circuit function and synapse organization, even before cognitive impairments can be detected.

We first determined whether elevated hippocampal activity could be detected in juvenile (6 week-old) mice in a well-studied animal model of AD-related amyloidosis, the APP/PS1 strain.^41^ To ensure that our results could not be attributed to plaque pathology or tau accumulation but rather to the effects of Aβ overproduction, we selected an age where neither cognitive impairments nor plaque deposition have been detected,^41^^,^^42^ but showing significant Aβ levels in the form of oligomers,^43^ coupled with synaptic dysfunction.^28^^,^^42^^,^^44^

**Figure 1:**
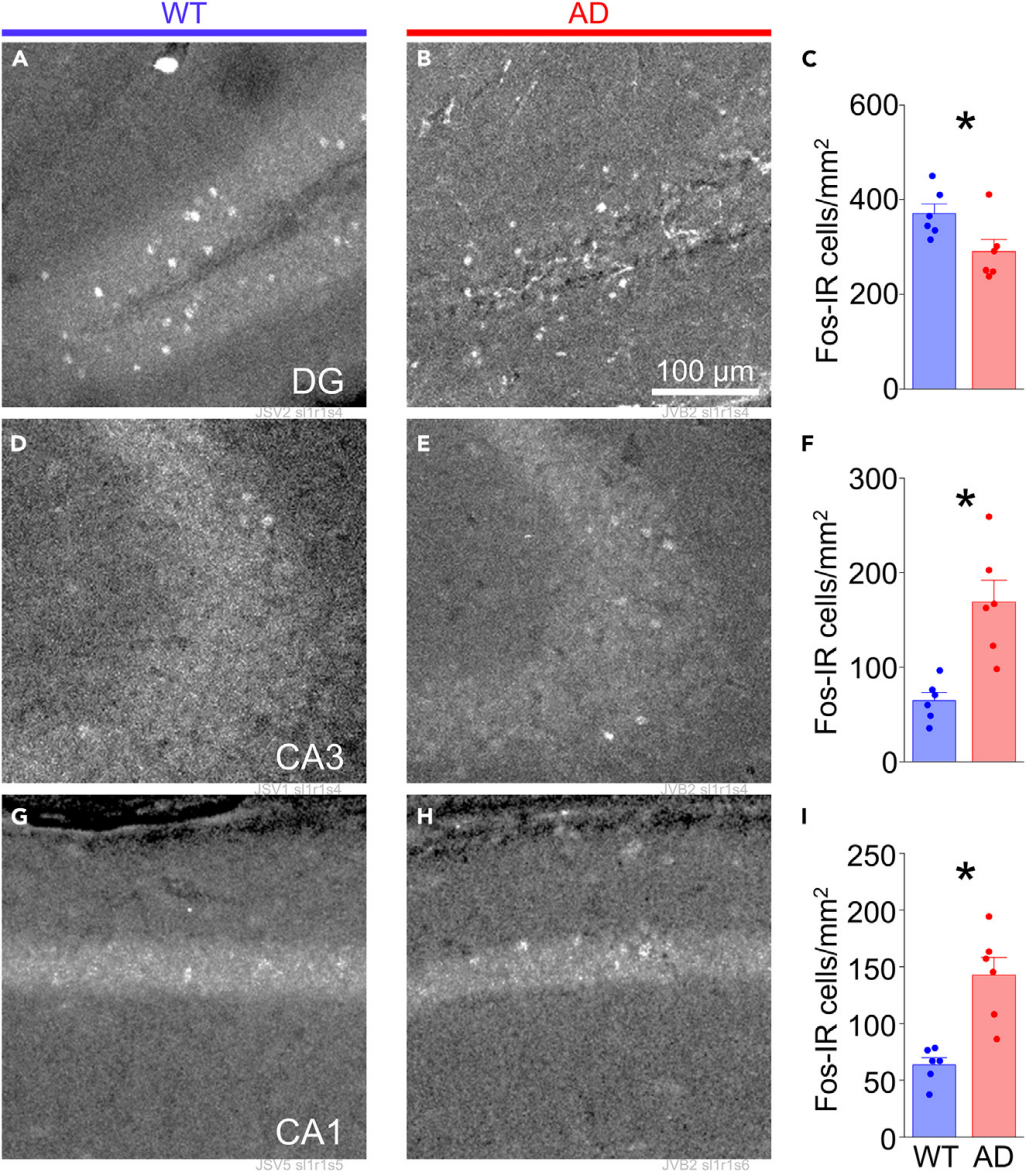
Basal Fos expression is elevated in hippocampus from juvenile APP/PS1 mice Fos-IR cells in dorsal hippocampus from APP/PS1 mice at 6 weeks of age. (A) Representative images of Fos-IR cells in DG from wild-type (WT) and (B) heterozygous (AD) mice. (C) Mean number of Fos-IR cells per section in DG for WT and AD mice (*n* = 6 mice each). Unpaired t(10) = 2.392; *p* = 0.0378). (D and E). Same as in (A, B) but for CA3. (F) Mean number of Fos-IR cells per section in CA3 for WT and AD mice (*n* = 6 mice each). Unpaired t(10) = −4.163; *p* = 0.0019). (G and H) Same as in (A, B) but for CA1. (I) Mean number of Fos-IR cells per section in CA1 for WT and AD mice (*n* = 6 mice each). Unpaired t(10) = −4.582; *p* = 0.0010). All bars represent mean + SEM; asterisks represent *p* < 0.05. Also see Figure S1.

We assessed abnormal hippocampal activity in juvenile APP/PS1 mice versus wild-type littermates by comparing expression of the activity-dependent IEG *c-fos*, a well-characterized indicator of elevated neural activity.^45^^,^^46^^,^^47^ Fos-immunoreactivity (Fos-IR) was assessed in the dorsal hippocampus from animals under resting, homecage conditions. Comparison of Fos-IR cells in three major subregions of the dorsal hippocampus revealed a significant difference in the frequency of Fos-IR cells in APP/PS1 transgenic mice (AD mice) compared to wild-type littermate controls (WT mice). APP/PS1 mice showed a 22% reduction in Fos-IR cells in the granular layer of the DG (WT 369.87 ± 20.70 vs. AD 289.76 ± 26.33 cells/mm^2^, *p* = 0.0378; Figures 1A–1C), but more than double the number of Fos-IR cells in the pyramidal cell layer (stratum pyramidale; SP) of CA3 and CA1 compared to age-matched wild-type littermates, differences that were highly significant (CA3: WT 64.41 ± 8.77 vs. AD 168.63 ± 23.45 cells/mm^2^, *p* = 0.0019; CA1: WT 63.68 ± 6.21 vs. AD 142.48 ± 16.03 cells/mm^2^, *p* = 0.001; Figures 1D–1I).

Electrode recordings and Ca^2+^ imaging studies have suggested that abnormal activity associated with Aβ overproduction might be enhanced by sleep or anesthesia.^12^^,^^48^ Thus, it was possible that we were underestimating Aβ-associated alterations of Fos-IR in transgenic mice. To test this possibility, we investigated whether levels of Fos-IR might be increased in mice exposed to isoflurane anesthesia. In contrast to prior studies indicating that anesthesia enhanced the difference in elevated Ca^2+^ transients for transgenic versus wild-type mice,^12^ we did not observe this to be the case under our experimental conditions for a small subset of animals (Figure S1). In CA1 and CA3, isoflurane exposure increased Fos-IR levels in both transgenic and wild-type mice, and reduced the difference in the DG. We conclude that Fos-IR is an imperfect indicator of neural activity,^49^^,^^50^ but may be a higher-order indicator of enhanced circuit activity, related to but not identical to other functional measurements.

Our data suggest a link between elevated Aβ/APP-processing and the emergence of abnormal activity in hippocampal circuits.^51^^,^^52^ These data are consistent with other studies showing hippocampal CA1 hyperexcitability in older mice from diverse models of amyloidosis^6^^,^^8^^,^^12^^,^^53^^,^^54^ and indicate that this abnormal activity is present significantly earlier than previously observed.

### FAPpost for quantitative synapse analysis

We hypothesized that Aβ exposure and abnormal circuit activity during development might be correlated with alterations in the number and distribution of hippocampal synapses in juvenile mice. Although the frequency and amplitude of miniature postsynaptic currents have been used to infer alterations in synaptic input, electrophysiological measurements cannot distinguish between inputs that occur in different dendritic compartments of a neuron, and soma-targeted whole-cell patch clamp recordings may be poorly suited to detection of inputs that lie in distal compartments due to dendritic filtering. Thus, we elected to examine whether Aβ overproduction or increased APP processing was linked to alterations in the size and distribution of synaptic inputs using the previously-characterized synapse marker FAPpost.^36^ FAPpost is a neuroligin-based tether attached to a fluorogen-activating protein that binds to a malachite green based fluorophore with a far-red emission and exceptional signal-to-noise in brain tissue.^55^^,^^56^

**Figure 2:**
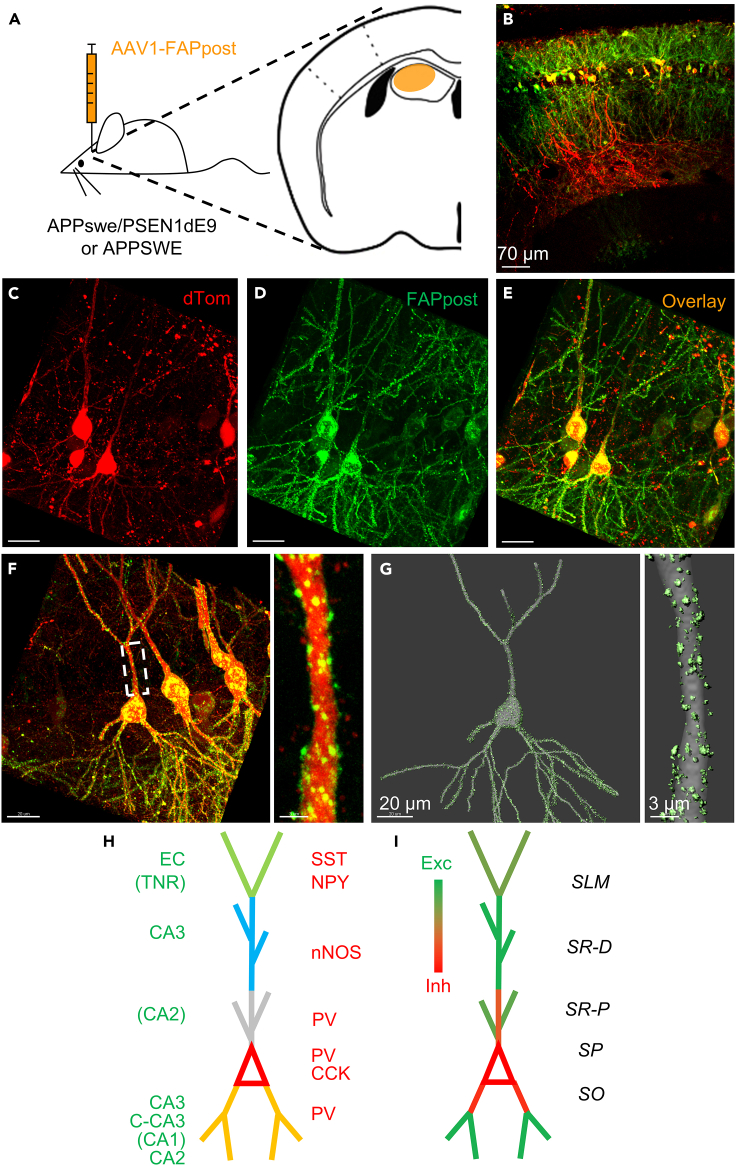
Fluorescence-based synaptic labeling to study compartment-specific synapse distribution on CA1 pyramidal neurons FAPpost was used for pan-synaptical labeling of hippocampal CA1 Pyr neurons. (A) Schematic of stereotaxic delivery of AAV1 encoding the FAPpost construct into mouse hippocampus. Cells are filled with dTomato (red) and FAPpost puncta (green) indicate postsynaptic sites. (B) Representative image with dTomato and FAPpost labeling across multiple neurons in dorsal CA1. (C–E) Representative fluorescence image showing multiple FAPpost-labeled CA1 Pyr. (F) Left: Representative image of CA1 Pyr used for 3D-reconstruction of FAPpost puncta across CA1 Pyr soma, proximal apical and basal dendrites. Right: Zoomed image of primary apical trunk from selected cell. (G) Left: Reconstructed CA1 Pyr (gray) with FAPpost post-synaptic puncta (light green); Right: Zoomed imaged of reconstruction of the primary apical dendrite trunk region. (H) Schematic of a CA1 pyramidal neuron (Pyr) showing laminar organization of pre-synaptic inputs, summarized from multiple studies.^25^^,^^58^^,^^59^^,^^60^^,^^61^^,^^62^^,^^63^ Different layers are marked with colors - apical tuft dendrites in *stratum lacunosum-moleculare* (*SLM*, green); apical dendrites in distal *stratum radiatum* (*SR-D*, blue); apical dendrites in proximal *stratum radiatum* (*SR-P*, gray); soma in *stratum pyramidale* (*SP*, red); basal dendrites in *stratum oriens* (*SO*, orange). Green and red labels indicate excitatory and inhibitory presynaptic inputs respectively - EC (entorhinal cortex), TNR (thalamic nucleus reuniens), CA3 (ipsilateral CA3), C-CA3 (commissural CA3), CA2 (ipsilateral CA2), CA1 (commissural and ipsilateral CA1), SST (somatostatin-expressing), NPY (neuropeptide-expressing), PV (parvalbumin-expressing), nNOS (neuronal nitric oxide synthase-expressing), CCK (cholecystokinin-expressing). (I) Schematic of excitatory:inhibitory synapse ratio across dendritic compartments of a CA1 Pyr as in (H). Green indicates exclusively excitatory and red exclusively inhibitory. Regions with mixed synaptic input are shaded proportionately. See also Figure S2 and Video S1.

We focused on CA1 Pyr neurons, as these neurons are the primary output of hippocampal circuits and also exhibited increased Fos-IR (Figure 2). FAPpost labeling showed punctate post-synaptic structures in the somata and dendritic arbors of CA1 Pyr neurons, consistent with its ability to label both excitatory and inhibitory synapses.^36^ We took advantage of the well-segregated inputs across CA1 basal dendrites, soma, and apical dendrites and tuft^25^^,^^57^ to quantitate compartment-specific alterations and identify potential input-specific synaptic changes (Figures 2H and 2I).

To determine whether Aβ/APP-fragments overproduction and increased Fos-IR were associated with synaptic changes on CA1 Pyr neurons, APP/PS1 and wild-type littermates were stereotaxically injected with an AAV-virus encoding the FAPpost construct (Figure S2; Video S1) into the dorsal hippocampus, and animals were perfused after 7–12 days of transduction. Tissue was sectioned and stained, and confocal image stacks were generated for volumetric reconstruction of labeled CA1 Pyr neurons. Three-dimensional reconstruction of neuronal processes enabled a more comprehensive estimate of the synaptic distribution on these neurons compared to spine analyses and Golgi staining,^28^^,^^29^^,^^30^^,^^31^^,^^64^ and indeed FAPpost puncta could be detected in non-spiny regions of the dendrite and soma.

### Inputs to the apical tuft of CA1 Pyr neurons are reduced in APP/PS1 mice

Synapse loss is prominent in animal models of AD, particularly in brain circuits that underlie navigation and short-term memory, including the entorhinal cortex and hippocampus.^28^^,^^29^^,^^65^^,^^66^ To determine whether reduced synapse density could be detected in the hippocampus of juvenile mice prior to the time of documented plaque deposition,^41^^,^^42^ we assessed the distribution of synapses at the apical tuft of CA1 Pyr neurons. This region receives dense excitatory input from entorhinal cortex and some midline thalamic inputs,^67^ as well as a smaller number of inhibitory synapses predominantly from somatostatin-expressing GABAergic neurons.^25^^,^^58^^,^^68^

**Figure 3:**
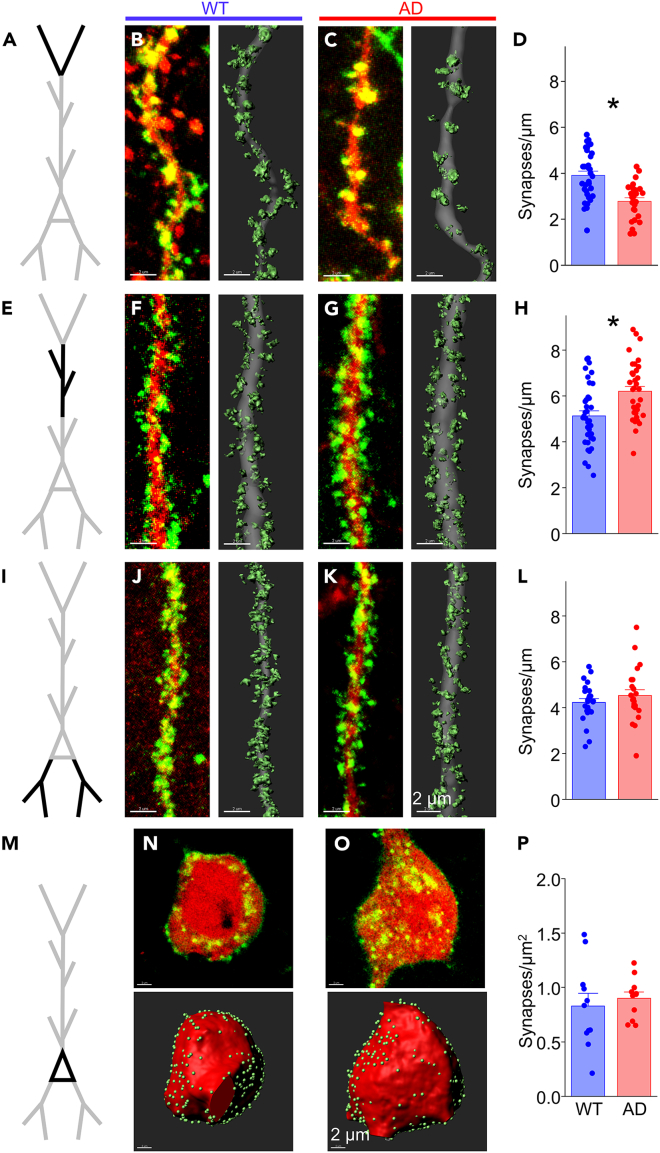
CA1 Pyr show dendrite-specific synapse gain and loss in juvenile APP/PS1 mice Synaptic density analysis in cellular compartments from CA1 Pyr neurons in APP/PS1 mice at 6 weeks of age. (A) Schematic of a CA1 Pyr with apical tuft dendrites (black) for analysis in (B–D). (B) Left: Fluorescent image showing FAPpost-marked synaptic puncta (green) and cell fill (red) on a tuft dendritic branch from a wild-type (WT) APP/PS1 mouse. Right: 3D-reconstruction of the fluorescence signal with synapses (light green) and dendrite (gray). (C) Same as in (B) but from a heterozygous (AD) APP/PS1 mouse. (D) Synapse density decreases at tuft dendrites in the AD group (red bar; 33 dendrites from 4 mice) compared to WT group (blue bar; 27 dendrites from 5 mice). Unpaired t(58) = 4.570; *p* = 2.597 x 10^−5^. (E–G) Same as in (A-C) but for apical dendrites in *SR-D* (black). (H) Synapse density increases at apical dendrites in the AD group (23 dendrites from 4 mice) compared to WT group (22 dendrites from 4 mice). Unpaired t(43) = −2.886; *p* = 0.0061. (I–K) Same as in (A–C) except for basal dendrites in *SO* (black). (L) No change detected in synapse density on basal dendrites in the AD group (35 dendrites from 5 mice) compared to WT group (37 dendrites from 5 mice). Unpaired t(70) = −3.441; *p* = 9.824 x 10^−4^. (M) Same as in (A) but for Pyr soma in *SP* (black). (N) Top: Fluorescent image showing FAPpost-marked synaptic puncta (green) and cell fill (red) on CA1 Pyr soma from a WT mouse. Bottom: 3D-reconstruction of fluorescence signal with synapses (light green) and soma (red). (O) Same as in (N) but from an AD mouse. (P) No change detected in somatic synapse numbers in the WT group (11 soma from 5 mice) compared to the AD group (11 soma from 4 mice). Unpaired t(20) = −0.555; *p* = 0.5848. All bars represent mean + SEM; asterisks represent *p* < 0.05. See also Figures S3 and S4.

Quantitative synapse analysis revealed a pronounced, 30% reduction in synapses on the apical tuft in juvenile APP/PS1 mice compared to wild-type littermates, a difference that was highly significant (WT 3.91 ± 0.18 vs. AD 2.79 ± 0.15 synaptic puncta/μm, *p* = 2.6 × 10^−5^; Figures 3A–3D; also see Figure S3 for animal-averaged puncta densities). Because the majority (∼85%) of synapses at the apical tuft are excitatory,^57^^,^^58^^,^^68^ it is likely that this reduction stems primarily from loss of excitatory inputs. These data indicate that Aβ/APP-fragments overproduction, abnormal hippocampal activity, and synapse loss are concurrent and can be detected in mice months before behavioral and cognitive deficits are detected.^42^^,^^69^

### CA3 inputs to CA1 Pyr neurons: apical and basal compartments

Aβ overproduction has been associated with alterations in synapse distribution in both the apical and basal dendrites of CA1 Pyr neurons.^27^^,^^28^^,^^29^ *In vitro*, Aβ (particularly Aβ_42_) can acutely enhance synaptic potentiation or depression, depending on the experimental paradigm.^14^^,^^22^^,^^70^^,^^71^ The interplay of these effects is poorly understood and has not been well-investigated in an intact network, particularly as they manifest over time.

The decrease in synapse density at the apical tuft suggested that other synapses across the somato-dendritic arbor might also be lost at this early developmental stage. We thus examined inputs to the apical and basal dendrites in APP/PS1 mice, which receive inputs from ipsilateral CA3 and mixed ipsi- and contralateral hippocampal Pyr neurons, respectively (Figure 2H).

Surprisingly, puncta density increased by ∼20% at apical dendrites in APP/PS1, a significant difference (WT 5.13 ± 0.22 vs. AD 6.20 ± 0.22 puncta/μm, *p* = 9.8 × 10^−4^; Figures 3E–3H), and basal dendrites showed no change (WT 4.23 ± 0.17 vs. AD 4.54 ± 0.25 puncta/μm, *p* = 0.302; Figure 3I–3L). In wild-type mice, puncta density was higher in CA1 apical (SR 5.13 ± 0.23 puncta/μm) than tuft (SLM 3.91 ± 0.19 puncta/μm; Tukey’s multiple-comparisons corrected *p* = 5.7 × 10^−5^; Figures 3B–3D, 3F, and 3H) and basal dendrites (SO 4.23 ± 0.17 puncta/μm, Tukey’s multiple-comparisons corrected *p* = 0.0083; Figures 3B–3D, 3J, and 3L), consistent with prior studies.^57^^,^^58^ The divergent alterations in synapse distribution indicate that both the location and presynaptic identity of synapses may be important variables in determining the effects of elevated Aβ/APP-processing.

### Somatic inputs are maintained in juvenile mice

Synapses onto the soma of CA1 Pyr neurons are almost exclusively inhibitory and arise predominantly from PV and CCK basket cells.^25^^,^^57^^,^^72^ We assessed whether somatic, putative inhibitory synapses might be influenced by Aβ/APP overexpression by quantitative analysis of FAPpost puncta on the soma of CA1 Pyrs (Figure 3M–3P). Although the density of somatic puncta varied across individual cells, we did not detect any difference in mean density from CA1 Pyr neurons from APP/PS1 and wild-type animals (WT 0.83 ± 0.12 vs. AD 0.90 ± 0.06 puncta/μm^2^, *p* = 0.585). Thus, synapse-specific alterations in juvenile mice are concentrated in the dendrites of CA1 Pyr.

### Tg2576 model of amyloidosis also shows early synapse redistribution in CA1

Animal models of AD typically elevate Aβ levels, since this is the main constituent of amyloid plaques. The APP/PS1 strain is a particularly aggressive animal model of amyloidosis, since overexpression of mutant presenilin facilitates cleavage of APP into Aβ_42_, the plaque-forming species of Aβ. In addition, presenilin 1 (PS1) may itself have synaptogenic effects,^73^ confounding the interpretation of synapse gain and loss in this strain. To determine whether an aberrant pattern of compartment-specific gain and loss might be common to other amyloidosis models, we selected Tg2576 mice, with overexpression of a human variant of APP carrying the Swedish mutations without additional PS1. These animals have elevated soluble Aβ compared to APP/PS1 mice but exhibit a slower progression of plaque pathology.^41^^,^^66^^,^^74^^,^^75^^,^^76^^,^^77^

Synapse distribution in CA1 Pyr neurons from Tg2576 transgenic mice was examined at 6 weeks of age when significant levels of Aβ species/oligomers can be detected in the brain^66^^,^^77^ (Figure S4). We focused on the synapse numbers in the apical compartments that were selectively affected in APP/PS1 mice. Puncta densities in the basal dendrites and soma of neurons from juvenile Tg2576 mice were not analyzed, as synaptic changes were not observed in these structures in the APP/PS1 strain. Consistent with analysis of the apical tuft in APP/PS1 mice, puncta density was significantly decreased in Tg2576 transgenic mice compared to wild-type littermates (Tg2576 WT 3.65 ± 0.14 vs. Tg2576 AD 3.24 ± 0.14 puncta/μm, *p* = 0.043; Figures S4A–S4D). The magnitude of this difference was less than observed in the APP/PS1 strain, possibly due to enhanced Aβ_42_ accumulation in the presence of PS1.

Similar to the APP/PS1 transgenic line, puncta density on the apical dendrites was also increased, though the magnitude of this increase was lower (Tg2576 WT 5.40 ± 0.21 vs. Tg2576 AD 6.07 ± 0.19 puncta/μm, *p* = 0.024; Figures S4E–S4H). The relatively modest synapse gain and loss across CA1 dendritic compartments from Tg2576 mice in comparison to age-matched APP/PS1 mice is consistent with its slower progression of amyloid pathology.^74^^,^^76^^,^^78^ Thus, we conclude that elevated Aβ/APP-processing initiates synaptic reorganization in the dorsal hippocampus of adolescent animals and may be a common feature of animal models of AD.

### Abnormal activity precedes synaptic changes

The co-occurrence of elevated Fos-IR and altered synapse distribution in juvenile mice made it difficult to infer which event preceded the other. For example, it is plausible that increased activity in CA3 might facilitate synapse addition at the apical dendrite of CA1 Pyr neurons, via enhancement of LTP,^23^^,^^71^ although there are other circuit inputs from entorhinal cortex, thalamus, inhibitory neurons, and the basal forebrain that might be affected in these animal models.^8^^,^^9^^,^^68^^,^^79^^,^^80^ Nonetheless, a temporal dissociation of elevated Fos expression and synaptic reorganization could suggest a sequence of events that might be relevant for future therapies.

**Figure 4:**
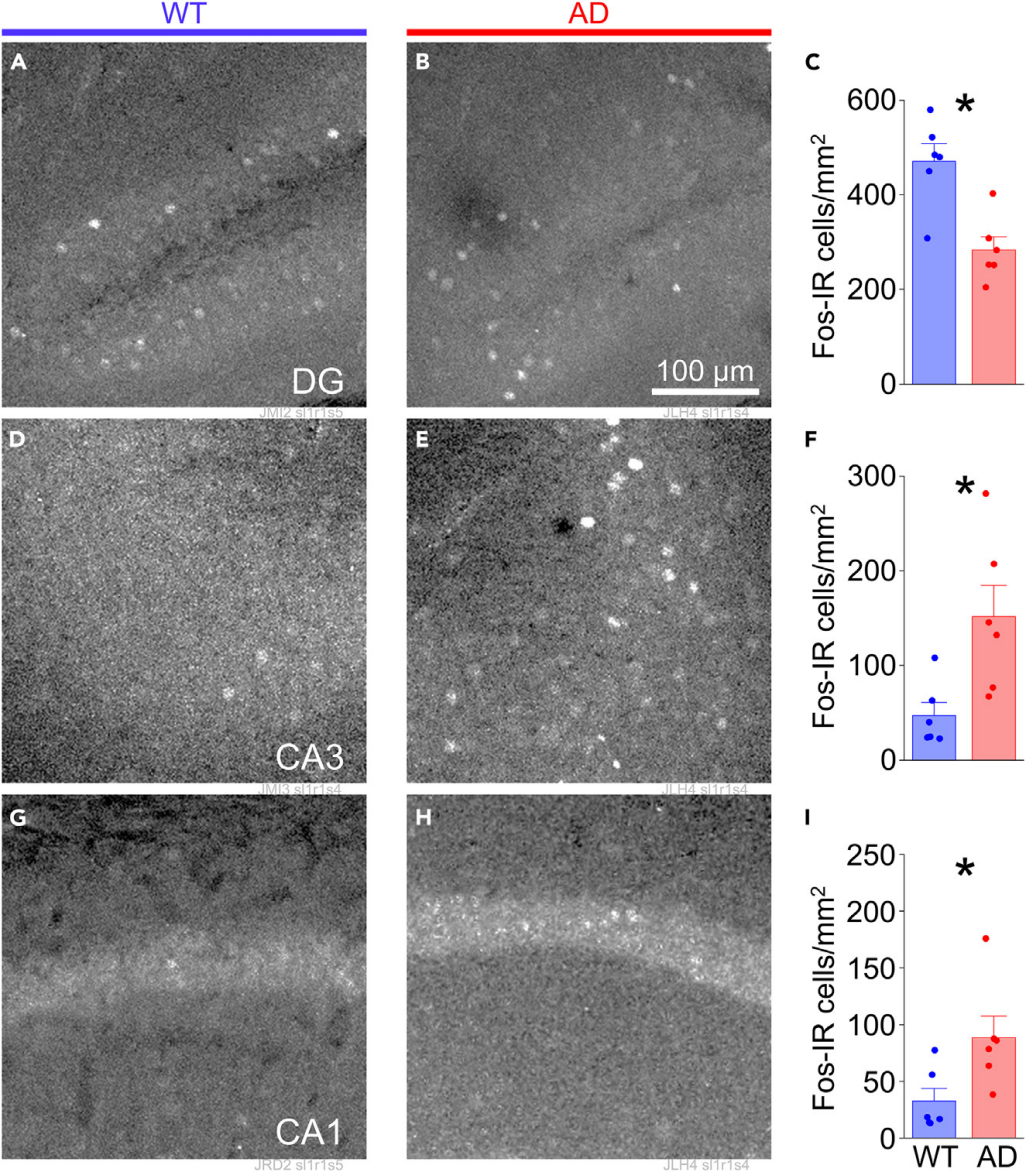
Basal Fos expression is elevated in hippocampus in APP/PS1 mice at weaning Fos-IR cells in dorsal hippocampus from APP/PS1 mice at 3 weeks of age. (A) Representative images of Fos-IR neurons in DG from wild-type (WT) and (B) heterozygous (AD) mice. (C) Mean number of Fos-IR cells per section in DG for WT (*n* = 6 mice) and AD (*n* = 6 mice). Unpaired t(10) = 4.030; *p* = 0.0024. (D and E) Same as in (A, B) but for CA3. (F) Mean number of Fos-IR cells per section in CA3 for WT (*n* = 6 mice) and AD (*n* = 6 mice). Unpaired t(10) = −2.909; *p* = 0.0156. (G and H) Same as in (A, B) but for CA1. (I) Mean number of Fos-IR cells per section in CA1 for WT (*n* = 6 mice) and AD (*n* = 6 mice). Unpaired t(10) = −2.530; *p* = 0.0299. All bars represent mean + SEM; asterisks represent *p* < 0.05.

To determine whether differences in synapse distribution preceded abnormal activity across the hippocampal network, we examined both Fos-IR and synapse distribution in APP/PS1 animals and their wild-type littermates at weaning (3 weeks of age; Figure 4). Immunohistochemical analysis indicated a significant decrease in Fos-expressing cells in the dentate gyrus (WT 470.56 ± 37.19 vs. AD 283.79 ± 27.66 cells/mm^2^, *p* = 0.0024; Figures 4A–4C) that was also accompanied by elevated expression in CA3 and CA1 (CA3: WT 47.15 ± 13.70 vs. AD 151.66 ± 33.21 cells/mm^2^, *p* = 0.0156; CA1: WT 32.55 ± 11.17 vs. AD 88.34 ± 19.01 cells/mm^2^, *p* = 0.0299; Figures 4D–4I). The absolute number of Fos-IR cells was higher in the dentate gyrus in weaned WT mice compared to juveniles, but it was lower in CA3 and CA1 in weaned mice (Figures 1 and 4). Thus, increase in Aβ/APP-fragment production is linked to Fos-IR at very early ages, during a time when hippocampal circuits are still maturing.^81^

**Figure 5:**
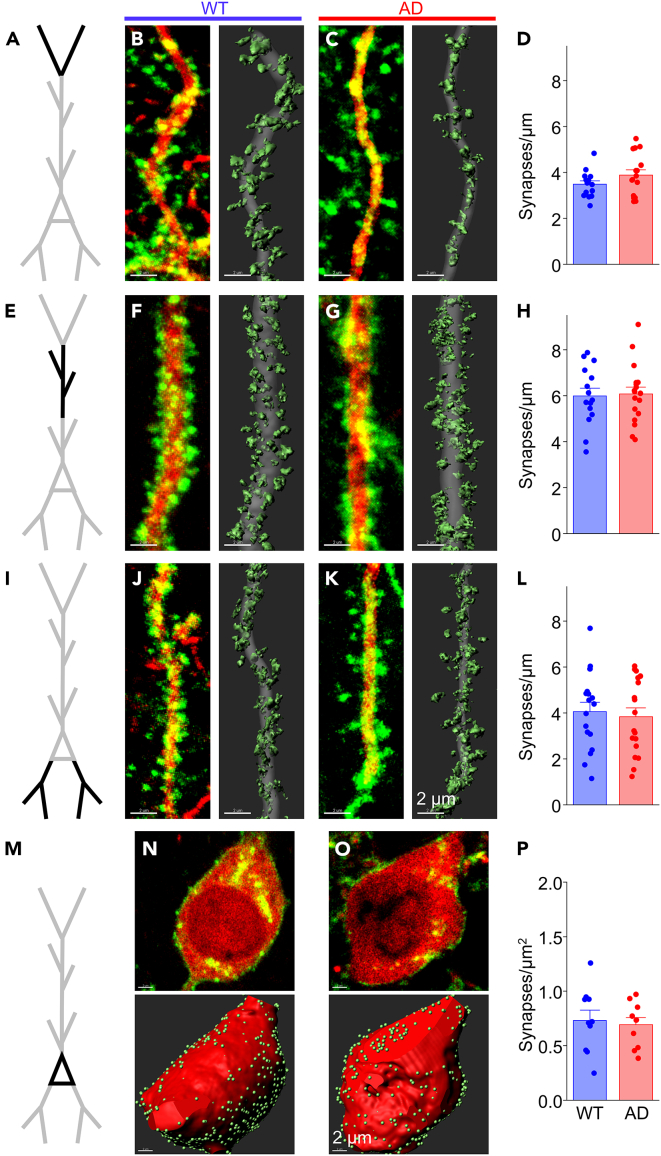
Synapse density across CA1 Pyr dendrites is similar in AD and WT mice at weaning Synaptic density analysis in cellular compartments in CA1 Pyr from APP/PS1 mice at 3 weeks of age. (A) Schematic of a CA1 Pyr with apical tuft dendrites (black) for analysis in (B–D). (B) Left: Fluorescent image showing FAPpost-marked synaptic puncta (green) and cell fill (red) on a tuft dendritic branch from a wild-type (WT) mouse. Right: 3D-reconstruction of the fluorescence signal with synapses (light green) and dendrite (gray). (C) Same as in (B) but from a heterozygous (AD) mouse. (D) Synapse density at tuft dendrites in the WT group (blue bar; 15 dendrites from 3 mice) compared to AD group (red bar; 16 dendrites from 3 mice). Unpaired t(29) = −1.445; *p* = 0.1593. (E–G) Same as in (A–C) but for apical dendrites in *SR-D* (black). (H) No change detected in synapse density at apical dendrites in WT group (15 dendrites from 3 mice) compared to the AD group (18 dendrites from 4 mice). Unpaired t(31) = −0.190; *p* = 0.8508. (I–K) Same as in (A–C) except for basal dendrites in *SO* (black). (L) No change in synapse density detected at basal dendrites in WT group (17 dendrites from 3 mice) compared to the AD group (18 dendrites from 4 mice). Unpaired t(33) = 0.400; *p* = 0.6918. (M) Same as in (A) but for Pyr soma in *SP* (black). (N) Top: Fluorescent image showing FAPpost-marked synaptic puncta (green) and cell fill (red) on CA1 Pyr soma from a WT mouse. Bottom: 3D-reconstruction of fluorescence signal with synapses (light green) and soma (red). (O) Same as in (N) but from an AD mouse. (P) No change in somatic synapse density in the WT group (10 soma from 3 mice) compared to the AD group (10 soma from 4 mice). Unpaired t(18) = 0.340; *p* = 0.7377. All bars represent mean + SEM; asterisks represent *p* < 0.05. See also Figure S3.

We next examined whether the compartment-specific differences in synapse distribution could be observed at this age (Figure 5; also see Figure S3 for animal-averaged puncta densities). Quantitative synapse analysis revealed that synapses at the apical tuft were not reduced in 3 week-old mice, but rather were modestly elevated though this difference was not significant (WT 3.49 ± 0.14 vs. AD 3.89 ± 0.23 puncta/μm, *p* = 0.159; Figures 5A–5D). Similarly, synaptic density at the apical dendrite was not altered (WT 5.99 ± 0.33 vs. AD 6.08 ± 0.30 puncta/μm, *p* = 0.851; Figures 5E–5H). No significant changes in the density of synapses across the basal dendrites (WT 4.06 ± 0.41 vs. AD 3.83 ± 0.38 puncta/μm, *p* = 0.692; Figures 5I–5L) or soma were detected (WT 0.73 ± 0.09 vs. AD 0.69 ± 0.06 puncta/μm,^2^ *p* = 0.738; Figures 5M–5P).

These data are consistent with a model where abnormal activity within the hippocampal circuit drives progressive alterations in function. In addition, these data indicate that elevated Aβ/APP-processing does not uniformly drive either synapse gain or loss even across a single target cell-type.

### Generalized synapse loss across the dendrites of aged APP/PS1 mice

Synapse loss is a hallmark of AD, where it has been observed both in human postmortem specimens,^19^^,^^20^^,^^82^ as well as in Aβ overproduction models including APP/PS1 mice.^28^^,^^29^ We verified that these synaptic phenotypes could also be observed with quantitative synapse analysis of CA1 Pyr neurons using FAPpost synapse labeling in mice at 12–14 months of age. Animals at this time-point display marked cognitive deficits as well as pervasive plaque pathology in multiple brain areas, including the hippocampus.^76^^,^^83^^,^^84^

**Figure 6:**
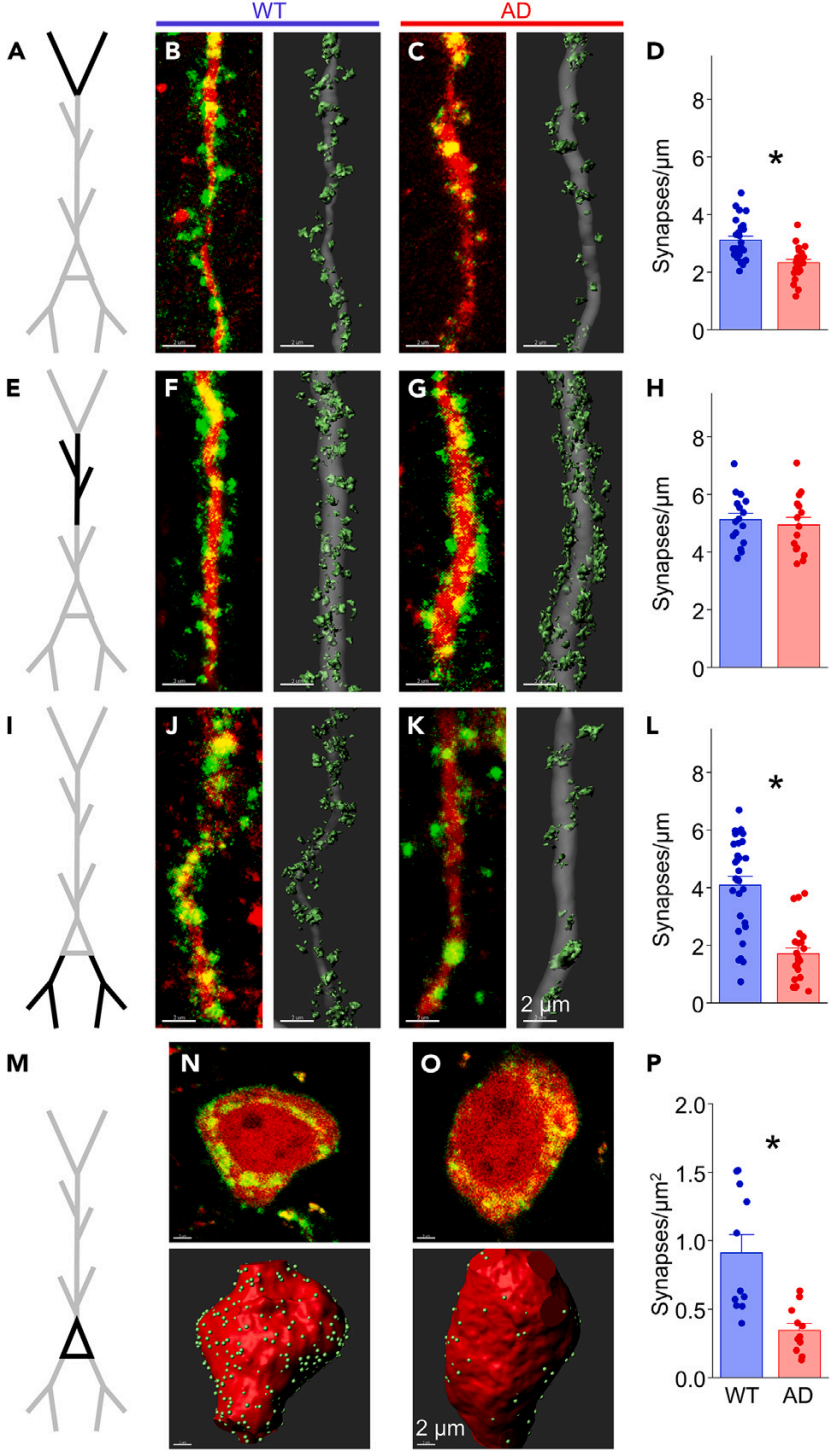
Synapse loss is widespread in CA1 Pyr neurons from aged mice Synaptic density analysis in cellular compartments from CA1 Pyr in APP/PS1 mice aged 12–15 months. (A) Schematic of a CA1 Pyr with apical tuft dendrites (black) for analysis in (B–D). (B) Left: Fluorescent image showing FAPpost-marked synaptic puncta (green) and cell fill (red) on a tuft dendritic branch from a WT mouse. Right: 3D-reconstruction of the fluorescence signal with synapses (light green) and dendrite (gray). (C) Same as in (B) but from an AD mouse. (D) Synapse density is decreased on tuft dendrites from the AD group (red bar; 22 dendrites from 4 mice) compared to the WT group (blue bar; 27 dendrites from 3 mice). Unpaired t(47) = 4.237; *p* = 1.049 x 10^−4^. (E–G) Same as in (A–C) but for apical dendrites in *SR-D* (black). (H) Synapse density is not altered at apical dendrites in the WT group (16 dendrites from 3 mice) compared to the AD group (15 dendrites from 3 mice). Unpaired t(29) = 0.532; *p* = 0.5989. (I–K) Same as in (A–C) except for basal dendrites in *SO* (black). (L) Synapse density is decreased at basal dendrites in the AD group (24 dendrites from 4 mice) compared to the WT group (31 dendrites from 5 mice). Unpaired t(53) = 6.182; *p* = 9.264 x 10^−8^. (M) Same as in (A) but for Pyr soma in *SP* (black). (N) Top: Fluorescent image showing FAPpost-marked synaptic puncta (green) and cell fill (red) on CA1 Pyr soma from a WT mouse. Bottom: 3D-reconstruction of fluorescence signal with synapses (light green) and soma (red). (O) Same as in (N) but from an AD mouse. (P) Somatic synapse density is decreased in the AD group (11 soma from 4 mice) compared to the WT group (11 soma from 5 mice). Unpaired t(20) = 3.914; *p* = 8.603 x 10^−4^. All bars represent mean + SEM; asterisks represent *p* < 0.05. See also Figures S3 and S5.

Analysis of puncta density at the apical tuft, the apical dendrite, the soma, and the basal dendrites revealed significant synapse loss in all compartments except for the apical dendrite (Figure 6; also see Figure S3 for animal-averaged puncta densities). In wild-type animals, tuft synapse density was lower in aged animals compared to 6 weeks of age (WT: aged 3.11 ± 0.13 vs. juvenile 3.91 ± 0.18 puncta/μm, Tukey’s multiple-comparisons corrected *p* = 0.002; Figure S5B), indicating an age-dependent synapse loss independent of amyloid pathology.^85^ The magnitude of synapse loss (∼25%) in APP/PS1 mice compared to age-matched wild-type littermates at the apical tuft (WT 3.11 ± 0.13 vs. AD 2.32 ± 0.12 puncta/μm, *p* = 1.0 × 10^−4^; Figures 6A–6D) was similar across these two ages. Also, tuft synapse density in aged APP/PS1 mice showed a non-significant decrease compared to juvenile animals (AD: aged 2.32 ± 0.12 vs. juvenile 2.79 ± 0.15 puncta/μm, Tukey’s multiple-comparisons corrected *p* = 0.091; Figure S5C), and both groups were significantly lower than weaned APP/PS1 mice (AD: weaned 3.89 ± 0.23 puncta/μm).

Aging did not alter synapse density in basal dendrites from wild-type mice (WT: aged 4.08 ± 0.30 vs. juvenile 4.23 ± 0.17 puncta/μm, Tukey’s multiple-comparisons corrected *p* = 0.926; Figure S5H). However, prolonged exposure to Aβ in aged APP/PS1 mice drove a marked, more than 2-fold reduction in synapse density in this compartment compared to juvenile and weaned APP/PS1 mice (AD: aged 1.71 ± 0.20 vs. juvenile 4.54 ± 0.24 puncta/μm, Tukey’s multiple-comparisons corrected *p* = 3.1 × 10^−10^; aged vs. weaned 3.83 ± 0.38 puncta/μm, Tukey’s multiple-comparisons corrected *p* = 2.7 × 10^−6^; Figure S5I). Compared to age-matched wild-type littermates, Aβ overexpression in aged animals was associated with a highly significant, 58% decrease in synapse density at the basal dendrites (WT 4.08 ± 0.30 vs. AD 1.71 ± 0.20 puncta/μm, *p* = 9.3 × 10^−8^; Figures 6I–6L). The increase in synapse density at the apical dendrite was not maintained in aged APP/PS1 animals (WT 5.13 ± 0.22 vs. AD 4.95 ± 0.26 puncta/μm, *p* = 0.599; Figures 6E–6H), suggesting that Aβ-associated synapse loss might be mitigated by early synapse gain in some dendritic compartment(s).

**Figure 7:**
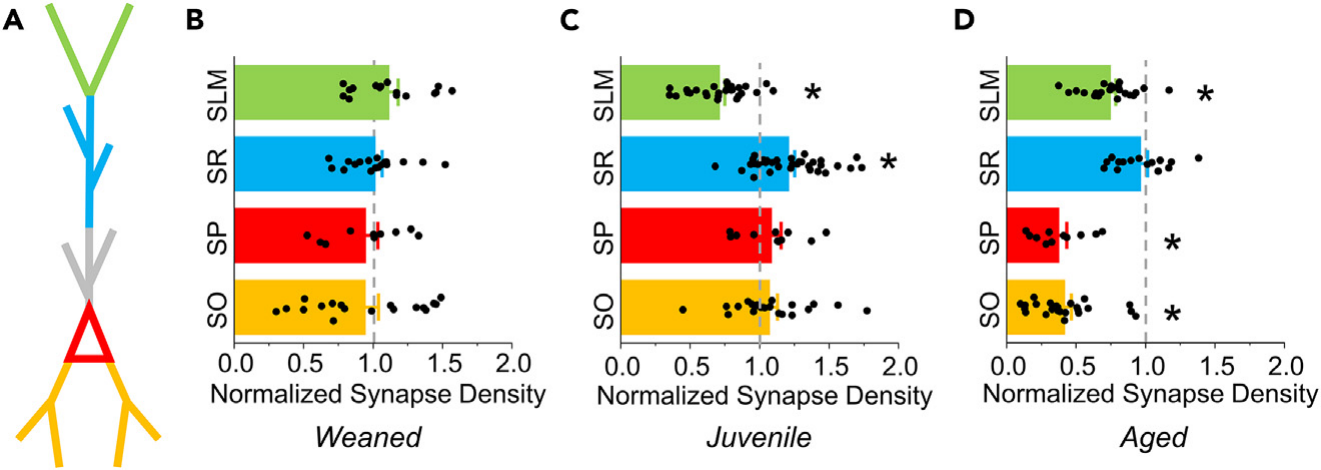
Summary of synapse density changes across age in APP/PS1 mice (A) Schematic of a CA1 Pyr highlighting different synaptic compartments: apical tuft in *SLM* (green), apical and oblique dendrites in *SR-D* (blue), apical dendrites in *SR-P* (gray), somata in *SP* (red), and basal dendrites in *SO* (orange). (B) Compartmental synapse densities from 3 week-old APP/PS1 transgenic mice are normalized to WT mean values (gray dashed line). (C–D) Same as in (B) but for 6 weeks- and 12–15 month-old mice respectively. All bars represent mean + SEM. Asterisks indicate synapse density distributions significantly different (*p* < 0.05) from WT based on t-test results from earlier figures. See also Figure S5.

Somatic synapse density also showed a more than 2-fold loss in APP/PS1 mice (WT 0.91 ± 0.13 vs. AD 0.35 ± 0.05 puncta/μm^2^, *p* = 8.6 × 10^−4^; Figures 6M–6P). Because somatic synapses are almost exclusively inhibitory, these results suggest that aging in the context of elevated Aβ/APP-processing drives a progressive loss of inhibition in CA1 Pyr neurons. A comparison of synaptic densities across age is summarized in Figure 7 (also see Figure S5).

## Discussion

Increasing experimental evidence indicates that hippocampal activity is altered prior to cognitive impairment and plaque detection in both animal models as well as human patients, suggesting that synaptic phenotypes may be present at early stages of overproduction of Aβ or APP-products. To evaluate early synaptic alterations in presymptomatic mouse models of amyloidosis, we assessed both activity-dependent gene expression and synaptic distribution in CA1 Pyr neurons using a quantitative fluorescence-based approach. Our results indicate that elevated levels of Aβ^43^^,^^66^^,^^77^ or APP-products is linked to elevated activity and compartment-specific synapse gain and loss in juvenile mice. Depending on synapse location, juvenile APP/PS1 mutant mice show both an increase in synapse density at the apical and oblique dendrites in the SR and a decrease in synapse density at the apical tuft, in the SLM. This statistically significant reorganization is preceded by abnormal and elevated levels of Fos-IR in CA3 and CA1, suggesting a link between circuit-level abnormalities in hippocampal activity and subsequent synaptic redistribution. Our data suggest that Aβ overexpression may initiate progressive circuit dysfunction, a sequence of synaptic reorganization events that is age-dependent.^86^

### Fos as an indicator of elevated activity

The IEG *c-fos* has been used to identify both brain regions and specific cells that are engaged by circuit activity during different behavioral and disease conditions.^46^^,^^47^^,^^87^ We observed that hippocampal Fos-IR was altered in transgenic mice even at weaning, preceding significant changes in synapse distribution. This suggests that synaptic reorganization in CA1 Pyr neurons is not driving abnormal activity. It is notable that both Fos-IR and synapse density experiments were carried out in animals taken from their home-cage, without specific stimuli that might enhance hippocampal activity. Although there may be other behavioral or brain-state conditions that drive abnormal activity - and enhance the relative difference between transgenic and wild-type mice – we conclude that basal levels of activity may be sufficient to drive synaptic reorganization. Other brain areas connected to the hippocampus, such as the entorhinal cortex, should be evaluated for early synaptic and activity changes.

Was the reorganization of synapses in CA1 Pyr neurons directly tied to increased Fos in a given neuron? Because Fos induction is linked to both bursting activity^88^ and synaptic input,^50^ it is difficult to determine the specific circumstances that precede its expression in an individual neuron. Anatomical reconstructions of CA1 Pyrs were not carried out in tandem with Fos-IR, and the number of Fos-IR neurons in immunolabeled sections was low, less than 5% of the total population within the Pyr cell layer. Thus, it is unlikely that our quantitative synapse analysis in CA1 Pyr neurons was restricted to the small fraction of Fos-IR cells detected in immunohistochemistry experiments. Although prior studies have identified Aβ-associated hyperexcitability in CA1 neurons, this abnormal activity was not easily detected by Ca^2+^ imaging or recording electrodes across a large population of neurons under awake conditions,^12^ suggesting that elevated hippocampal Fos-IR in transgenic mice might not directly correspond to spiking activity over short time intervals. Indeed, it remains unclear whether the population of Fos-IR neurons are maintained over time or whether they transit in and out of an activated population (see for example Lee et al.^87^). In addition, intracellular signaling pathways controlling Fos activation may also be altered in transgenic mice. Thus, while we are unable to pin down the exact sources of increased Fos-IR activity, our data are consistent with the idea that a small subpopulation of highly-active cells in CA3 and CA1 hippocampal subfields might have been missed in prior studies focused on neuronal recordings.

We conclude that elevated network activity across the hippocampus may be a general property of Aβ overproduction models, particularly in young mice. Future experiments that record hippocampal activity, both during active states and also during sleep, may reveal specific processing deficits associated with hippocampal function in animal models of amyloidosis. It will also be interesting to examine how baseline differences in number of Fos neurons in AD models may impact neuronal recruitment into hippocampal engrams.

### Synaptic reorganization is similar in APP/PS1 and Tg2576 strains

Prior studies examining synaptic distribution or dysfunction have typically focused on one of many available transgenic strains with Aβ overproduction.^28^^,^^42^^,^^43^^,^^89^^,^^90^^,^^91^ It has been unclear whether specific phenotypes are core features of disease progression or are idiosyncratic properties of a given strain. Importantly the opposing changes in tuft and apical synapse density we identified were conserved in both strains of mice. These changes were more pronounced in the APP/PS1, consistent with the slower progression of amyloid pathology in Tg2576 mice.^74^^,^^76^ The inclusion of mutant PS1 in the APP/PS1 strain biases APP cleavage toward formation of longer Aβ peptides that have broad effects on synaptic function and are linked to more rapid disease progression. In addition, mutant PS1 and other APP proteolytic products may also contribute to change in synaptic numbers.^73^^,^^92^ Our finding that hippocampal activity and synapse densities are similarly altered in both strains indicates that our results may be applicable to other mouse models of amyloidosis as well as human disease.

### Input identity of synaptic gain and loss

Because fluorescence-based approaches can reconstruct the 3-dimensional architecture of the dendrite, our results present a more comprehensive picture of inputs onto CA1 Pyr in comparison to spine-based or Golgi-stained tissue analyses.^28^^,^^29^^,^^31^ In general, the synaptic densities reported here were consistent with EM-based estimates.^57^^,^^58^ Although there are other classes of synapse-labeling reagents,^33^^,^^34^^,^^93^ FAPpost labels both excitatory and inhibitory synapses^36^ and is advantageous for neuron-scale quantitative analysis.

We used synaptic location along the neuron to infer input identity (Figure 3). Our study only indirectly assessed excitatory versus inhibitory synaptic changes, using the dendritic location of synapses to infer neurotransmitter identity. Somatic inputs – which are almost exclusively inhibitory^25^^,^^57^ onto CA1 Pyr neurons were not altered in juvenile animals, at the same time when there were clear effects in other dendritic compartments. Inhibitory synapses comprise ∼15% of synapses at the apical tuft.^57^^,^^58^ Indeed, a main source of inhibition at the apical dendrite arises from somatostatin neurons, and this is not altered until animals are more than 4 months of age.^68^ Because we observed a synaptic loss of nearly 30% in tufts – greater than the documented fraction of inhibitory synapses in this compartment – we conclude that it cannot be only inhibitory synapses that are eliminated in these mouse models of amyloidosis.

Inputs to the apical dendrite in the SR primarily arise from CA3 Pyr, with only a small minority arising from inhibitory synapses (2–3%).^57^^,^^58^ Thus, the increase in puncta density in this compartment likely represents an increase in excitatory inputs to CA1 Pyr neurons. Indeed, prior studies in both the Tg2576 and APP/PS1 strains are consistent with our findings, showing an increased spine density at the apical dendrite receiving CA3 inputs as early as 1 month of age.^27^^,^^28^

### Location-specific synapse gain and loss

Why was synapse distribution increased in one compartment but reduced in another? Because we directly compared synapse density in specific compartments for wild-type and transgenic mice, it is unlikely that the differences observed at the apical tuft can be attributed to a systematic problem with FAPpost transport to distal dendrites. We cannot rule out the possibility that intracellular transport may be compromised in transgenic mice with Aβ overexpression.^94^ However, we did not observe a decrease in FAPpost puncta density based on distance from the soma at the apical dendrite. Future experiments to identify the specific class of inputs in these dendritic compartments will be useful in identifying circuit-specific alterations associated with hippocampal dysfunction.

Our data suggest that presynaptic identity might be an important variable in alterations of synapse distribution, where tuft synapses arise from entorhinal cortex and midline thalamus, and synapses along the apical dendrite come from CA3 inputs. Presynaptic identity might confer different molecular properties to these synapses or might be linked to different levels of afferent activity. Importantly, even excitatory synapses in different compartments of CA1 Pyr neurons exhibit distinct plasticity rules,^95^^,^^96^^,^^97^^,^^98^ and Aβ itself can interact with synapse-specific proteins, such as nicotinic acetylcholine receptors^99^ and different glutamate receptor subunits^100^^,^^101^^,^^102^ that may lead to selective susceptibility.^103^ It is also possible that Aβ overproduction is important for synaptic pruning during development, which may be altered in a compartment-specific manner. However, prior studies suggest that Aβ enhances pruning,^44^ contrary to the increase in synapse density observed in the apical dendrite.

Notably, neurons in entorhinal cortex have 5– to 6-fold higher firing rates than CA3 neurons,^104^ and Aβ production can be enhanced by increased neural activity/firing.^17^^,^^105^^,^^106^^,^^107^^,^^108^ Consistent with this, studies across different mouse models, including the APP/PS1 and Tg2576 strains, show that amyloid plaques are preferentially localized at the tuft in the SLM compared to the SR, consistent with the hypothesis that Aβ may be produced/accumulated at a higher rate near the tufts. The increase in synaptic density in the apical dendrite of CA1 Pyr neurons may be directly related to Aβ production at this synapse or could be a downstream consequence of Aβ effects on intrinsic excitability.^109^ Changes in the activity of other brains areas^9^^,^^110^ that were outside the scope of this study may initiate changes in hippocampal activity and synapse distribution.

Aβ has a complex and bidirectional effect on hippocampal LTP where lower levels of Aβ can enhance synaptic potentiation and higher levels cause impairment.^14^^,^^23^^,^^111^^,^^112^ Thus, compartment-specific differences in Aβ production might drive opposite changes in synaptic plasticity, ultimately manifesting as differences in synapse density. Aβ effects on inhibitory synapses have also been documented,^113^ and these synapses are particularly diverse in pre- and postsynaptic composition. The molecular cascades that lead to synapse-selective alterations, either directly or indirectly and at excitatory or inhibitory synapses, will be of great interest.

### Conclusion

Our results demonstrate that Aβ/APP-products drive broad and input-specific synapse reorganization during early stages of amyloidosis. Although Aβ and plaque formation has been associated with synapse loss,^114^ particularly at later stages of AD, our data indicate that synapse gain may also occur especially early in the disease. Our study also focused on alterations in synapse densities without addressing potential Aβ-related changes in size and strength of individual synapses,^115^ since the FAPpost marker was poorly suited to assess features of synaptic architecture. Longitudinal studies to examine changes in neural excitability and fine-scale synaptic measurements at brain scale across the age span will be informative in understanding how Aβ production is related to progressive cognitive decline.

Although APP overexpression in mouse models does not recapitulate the diversity of pathological and clinical features observed in AD,^116^^,^^117^ analysis of Aβ-related synaptic alterations may provide helpful insights into early stages of disease and potential treatment objectives. Overall, these data support the idea that presymptomatic AD may be a distinct phase of the disease with opportunities for targeted intervention.

### Limitations of the study

Our study is unable to address whether a direct correlation exists between differences in Aβ levels between CA1 layers and the compartment-specific synaptic changes observed, especially in young animals that do not display plaque pathology. Sensitive biochemical measurements of Aβ species and oligomerization – immunoblotting, ELISA, and/or mass spectroscopy – do not provide sufficient spatial resolution that would enable analysis of compartment-specific deposition. Thus, novel Aβ-oligomer specific labeling strategies coupled with advanced imaging methods such as super-resolution and expansion microscopy will be needed to reveal the relationship between local Aβ levels and synapse density. In addition, it will be interesting to study whether the compartment-specific effects arise from interplay between unique effects of different Aβ species, and other proteolytic products of APP or APP overexpression itself that may have unique synaptic effects independently of Aβ.^73^^,^^92^^,^^118^

## STAR★Methods

### Key resources table

**Table:** 

REAGENT or RESOURCE	SOURCE	IDENTIFIER
**Antibodies**		

Guinea pig anti-*c*-Fos	Synaptic Systems	#226003; RRID: AB_2231974
Goat anti-guinea pig Alexa Fluor 647	Thermo Fisher Scientific	#A-21450; RRID: AB_141882

**Bacterial and virus strains**		

AAV1-hSyn-Igkappa-myc-FAPdL5-POST-T2A-dTomato-WPRE-bGH	Penn Vector Core	Addgene #105981; RRID: Addgene_105981

**Chemicals, peptides, and recombinant proteins**		

Paraformaldehyde	Sigma-Aldrich	#441244
10X PBS	Alfa Aesar	#J62036
Triton X-100	Sigma-Aldrich	#T8787
Sucrose	Sigma-Aldrich	#S0389
Malachite green T-Carb (MG-TCarb)	Molecular Biosensor and Imaging Center (MBIC), CMU	Manufactured in house. https://doi.org/10.3389/fncel.2017.00337
Donkey serum	Sigma-Aldrich	#D9663
Isospire (isoflurane)	Dechra	https://www.dechra-us.com/our-products/us/companion-animal/dog/prescription/isospire-isoflurane-inhalation-anesthetic
Ketofen (ketoprofen)	Zoetis	https://www.zoetisus.com/products/equine/ketofen
Vectashield Plus antifade mounting media	Vector Laboratories	#H-1900

**Experimental models: Organisms/strains**		

Mouse: APP/PS1	Jackson Laboratory	#034829-JAX; RRID: MMRRC_034829-JAX
Mouse: Tg2576	Taconic Biosciences, Inc.	#1349

**Software and algorithms**		

Zen	Zeiss	https://www.zeiss.com/microscopy/en/products/software/zeiss-zen.html
Imaris 8.4.2	Oxford Instruments	RRID:SCR_014212; https://imaris.oxinst.com/packages
OriginPro 2022	Origin Lab	RRID:SCR_007370; https://www.originlab.com/index.aspx?go=PRODUCTS/Origin
FIJI	NIH	RRID:SCR_002285; http://fiji.sc

### Resource availability

#### Lead contact

Requests for further information, resources and reagents should be directed to and will be fulfilled by the lead contact, Alison Barth (albarth@andrew.cmu.edu).

#### Materials availability

This study did not generate any new unique reagents.

#### Data and code availability

• All data reported in this paper will be shared by the lead contact upon request.
• This paper does not report original code.
• Any additional information required to reanalyze the data reported in this paper is available from the lead contact upon request.

## Experimental model and study participant details

Both male and female mice were used for all analyses. B6;C3-Tg(APPswe, PSEN1dE9)85Dbo/MMjax (#034829-JAX; Jackson laboratory) heterozygous and wild-type mice (referred in text as APP/PS1 strain) of both sexes were used at the following ages - P21, P41-43 and 12-15 months - for synaptic and Fos analyses. For a subset of experiments, B6;SJL-Tg(APPSWE)2576 Kha (#1349; Taconic Biosciences, Inc.) heterozygous and wild-type mice (referred in text as Tg2576 strain) of both sexes at age P41-42 were used for synaptic analyses. All experimental procedures were conducted in accordance with the NIH guidelines and approved by the Institutional Animal Care and Use Committee at Carnegie Mellon University. Animals were separated by sex and housed in groups of up to 4 mice/cage.

Fos expression was measured in mice housed in their home-cages under resting conditions and is sensitive to experience. To avoid handling-related Fos-induction, we were careful to house WT and APP/PS1 animals together and avoid changes in caging 2–3 days prior to perfusion. Animals were perfused at the same time of day between 12 and 1 p.m., and animals from the same cage were perfused within 15 min of each other to ensure uniformity to prevent Fos translation and accumulation.^49^^,^^119^ For Fos-IR quantification in isoflurane-anesthetized mice, anesthesia was induced with 3–4% beginning at noon, and tested with the absence of a paw-pinch reflex. Animals were kept in a 1% isoflurane level for 2 h before perfusion.

### Method details

#### Stereotaxic surgeries

Stereotaxic surgeries were performed in mice at P14, P30 and 12–15 months respectively for age-based analysis. Animals were perfused 7–12 days later. For FAPpost labeling, ∼150 nL of AAV1-hSyn-Igkappa-myc-FAPdL5.POST-T2A-dTomato-WPRE-bGH virus (Addgene #105981, Penn Vector Core, Philadephia, PA; titer 7.56 × 10^12^ GC/mL)^36^; was injected into two sites in the right dorsal CA1 (from bregma: x = −1.5/1.9, y = −2.0/2.3, z = −1.2/1.35 mm from pial surface) in isoflurane-anesthetized mice (Figure 2). Injections were carried out through two separate craniotomies using a Nanoject II (Drummond Scientific Company; Broomall, PA). Post-operative analgesia was provided using *s.c.* ketoprofen.

#### Reagents

All chemicals were purchased from Sigma-Aldrich (St Louis, MO) unless otherwise indicated.

#### Tissue processing and histology

At midday, mice were deeply anesthetized with isoflurane and transcardially perfused using 20 mL 1X PBS (pH 7.4) followed by 20 mL 4% paraformaldehyde in 1X PBS (4% PFA; pH 7.4). To avoid handling and stress-related changes in Fos expression, mice were individually or dually housed 3–4 days before perfusion and euthanized within 15 min of handling onset. Brains were then removed and post-fixed overnight at 4°C in 4% PFA before transfer into 30% sucrose in 1X PBS. After osmotic equilibration, ∼50 μm-thick free-floating brain sections were collected in 1X PB using a freezing-microtome (Leica Biosystems, Wetzlar, Germany).

#### c-Fos immunohistochemistry and quantitation

Hippocampal sections were immunostained with Fos antibody for assessing altered activity. Briefly, the sections were blocked (10% donkey serum, 0.5% Triton X-100, and 1X PBS) for 2 h, and then incubated for ∼40 h at 4°C with guinea pig anti-Fos primary antibody (1:400 in blocking solution; Synaptic systems, Goettingen, Germany; #226003). Slices were rinsed five times with 0.5% PBST, and then incubated with Alexa Fluor 647 anti-guinea-pig secondary antibody (1:500 dilution in 1X PBS; Thermo Fisher Scientific, Waltham, MA; A-21450). Sections were washed 5 times in 1X PBS and then mounted on glass microscope slides with Vectashield Plus antifade mounting media (Vector laboratories, Burlingame, CA).

Confocal imaging and analysis were carried out blind to genotype. Fos-immunoreactive (Fos-IR) cells were quantified using the “Cell Counter” plugin in FIJI (NIH)^120^ in either CA1, CA3 or DG subregions from four-five 50 μm-thick sections from the dorsal hippocampus (typically the left hemisphere) sampled as every alternate section between 1.46 and 2.3 mm posterior to bregma.^121^ Counts in the granular areas of individual sections were normalized to the respective sub-regional granular area, and expressed as cells/mm^2^. These normalized counts were then averaged across all sections from an individual animal, and were used for final statistical testing and plotting. Counts were repeated by two independent blinded observers.

#### FAPpost staining

Free-floating brain sections containing dTom-expressing cells were washed using 1X PBS before 30 min room temperature incubation with malachite green (MG) T-carb dye (300 nM in 1X PBS.^36^ Stained sections were then rinsed five times with 1X PBS before mounting on glass microscope slides with Vectashield Plus antifade mounting media. Sections with dorsal CA1 pyramidal neurons with punctate FAPpost labeling and bright dTomato cell-fills were mounted on slides for confocal imaging.

#### Image acquisition

Sections were imaged using the LSM 880 AxioObserver microscope (Carl Zeiss; Oberkochen, Germany). For Fos-IR tissue, a 10X air objective was used with the pinhole set at 1.0 Airy disk unit for excitation λ633 and detection λ633-695. Resultant image sizes were either 1024 × 1024 or 2048 × 2048 pixels to capture the entire dorsal hippocampus, and the zoom factor was set at 0.6 with a 4.75 μm Z-step size, resulting in voxel dimensions of 1.38 × 1.38 × 4.75 μm (X, Y, and Z).

For FAPpost, a 63X oil-immersion objective lens (Plan-Apochromat, NA 1.40, oil) with the pinhole set at 1.0 Airy disk unit was used for all fluorescence channels (dTomato: excitation λ561, detection λ561-625; FAPpost: excitation λ633, detection λ633-695). FAPpost image size was 1024 × 1024 pixels, and the zoom factor was set at 0.9 with a 0.3 μm Z-step size, resulting in voxel dimensions of 0.146 × 0.146 × 0.3 μm (X, Y, and Z). Resultant image stacks were typically 149.5 × 149.5 × ≤ 40 μm.

Optimal laser intensities and gains for each channel were set to avoid pixel under-or over-saturation for each field of view independently. Images were stored in .czi format for subsequent analyses.

#### Digital reconstruction

“.czi” image files were imported into Imaris version 8.4.2 equipped with the Filament Tracer plugin (Oxford Instruments) for semi-automated digital analysis.^36^ Well-isolated FAPpost-labeled cells and dendrites with a clear dtomato fill and defined puncta were selected from CA1 of dorsal hippocampus for reconstruction and analysis. Basal dendrites were not divided into primary and higher order branches since the vast majority (>95%) of the basal dendrite traces comprised higher-order. Apical dendrite analysis only focused on higher-order dendrites, not including the apical trunk. Due to the poor segregation of different excitatory and inhibitory inputs to the proximal apical branches (trunk; gray dendritic compartment in Figure 2H), we did not to analyze this compartment.^57^ Only dendrites that could be continuously traced for ≥20 μm were used for synapse measurements and synapse density is reported as linear density. Cell somata were fully encompassed within the optical stack for somatic synapse quantification expressed as synapses per μm.^2^ We have used puncta density and synapse density interchangeably throughout the manuscript.

The dTomato cell fill was used to create a 3D cell surface rendering using a combination of surface and filament objects. For cell and neurite reconstruction from selected dendrites, FAPpost puncta were first reconstructed as 3D structures using “surface objects” (to outline puncta borders). To ensure that all puncta were detected, fluorescent signal was enhanced to enable visualization of both bright and weak puncta. Subtraction of background fluorescence was performed by masking cell-associated fluorescence. Due to imaging limitations, only puncta larger than 3 voxels (∼0.02 μm^3^) were counted, potentially undercounting very small synapses below this detection threshold. Large puncta that potentially reflected smaller, fused synapses were separated into multiple objects with an estimated 0.5 μm diameter using the “split touching objects” function in Imaris. Thus, large puncta were potentially separated into multiple smaller synapses, a process that could increase the absolute number of detected synapses. Puncta were digitally associated with the plasma membrane if their edges lay within 0.5 μm from the soma cell surface or 1 μm from the surface dendritic shaft (to account for dendritic spines). Puncta 0.5 μm below the cell surface were attributed to cytosolic signals and not included for analysis.

### Quantification and statistical analysis

Synaptic analyses were carried out blind to animal genotype. All statistical tests and graphing were performed with OriginPro (version 2022; OriginLab Corporation, Northampton, MA). Synapse densities in the text are reported as mean ± SEM. Differences across genotype in age-matched animal groups were tested using unpaired t-tests. Differences across age or dendritic compartments were corrected for multiple comparisons using Tukey’s *post hoc* test following an ANOVA test. *p* < 0.05 was considered for assessing statistical significance. Power analyses were not carried out. Statistical analyses were not segregated by sex as a biological variable due to insufficient statistical power and are beyond the scope of this study, although we note that neuroanatomical and behavioral deficits may be enhanced in young male mice of the APP/PS1 strain.^122^

## Supplemental information

Document S1. Figures S1–S5

Video S1. FAPpost-labeled CA1 Pyr neuron from a juvenile (6-week old) APP/PS1 heterozygous mouse, related to Figure 2
