# Supplementary material for: Early hippocampal hyperexcitability and synaptic reorganization in mouse models of amyloidosis
Source: iScience. 2024 Aug 2;27(9):110629. doi: 10.1016/j.isci.2024.110629 (PMC11388185; doi:10.1016/j.isci.2024.110629)
Supplement: Document S1. Figures S1–S5 [file mmc1.pdf]

iScience, Volume *27*

Supplemental information

Early hippocampal hyperexcitability and synaptic reorganization in mouse models of amyloidosis

Ajit Ray, Iulia Loghinov, Vijayalakshmi Ravindranath, and Alison L. Barth

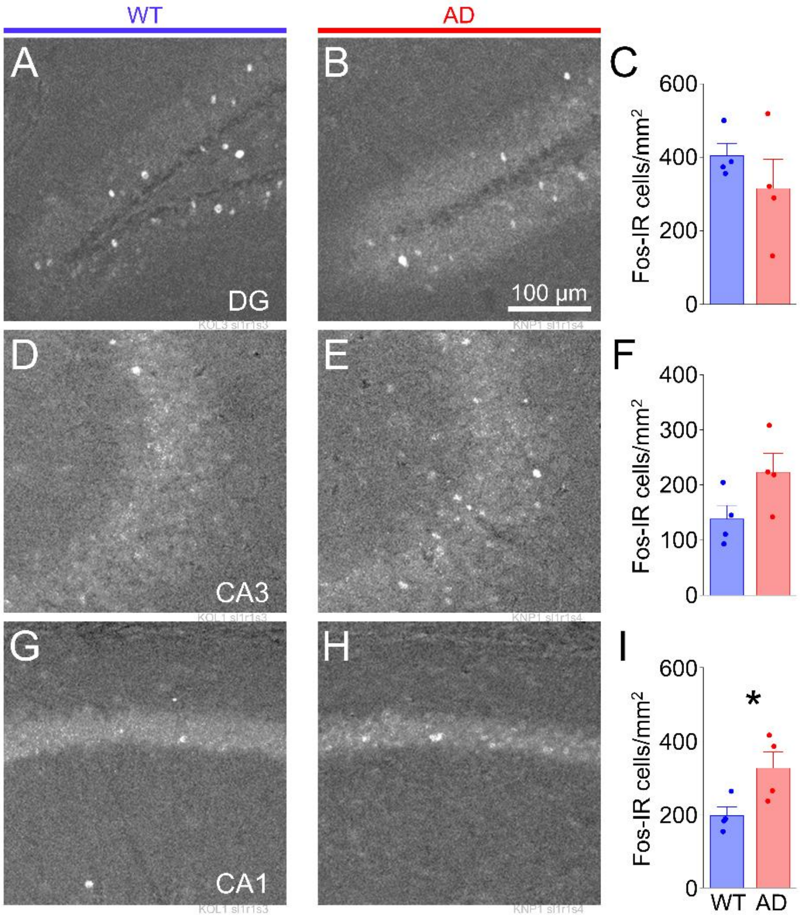

Figure S1. Isoflurane anesthesia enhances basal Fos expression, *related to Figure 1*.
Fos-IR cells in hippocampal regions from isoflurane-anesthetized, juvenile APP/PS1 mice.
(A) Representative images of Fos-IR cells in DG from wild-type (WT) and (B) heterozygous (AD) mice.
(C) Mean number of Fos-IR cells per section in DG from for WT and AD mice (n = 4 mice each). Unpaired $t(6) = 1.039$; $p = 0.3389$.
(D, E) Same as in (A, B) but for CA3.
(F) Mean number of Fos-IR cells per section in CA3 for WT and AD mice (n = 4 mice each). Unpaired $t(6) = -2.023$; $p = 0.0895$.
(G, H) Same as in (A, B) but for CA1.
(I) Mean number of Fos-IR cells per section in CA1 for WT and AD mice (n = 4 mice each). Unpaired $t(6) = -2.578$; $p = 0.0419$.
All bars represent mean + SEM; asterisks represent $p < 0.05$.

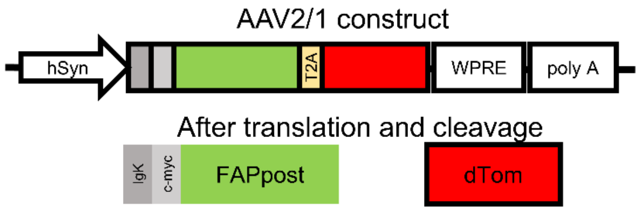

Figure S2. FAPpost construct design, *related to Figure 2*.
Schematic showing virally-transduced FAPpost construct driven by the human synapsin promoter. After translation, the T2A side is auto-cleaved to produce FAPpost protein trafficking to synapses and dTomato cell fill [S1].

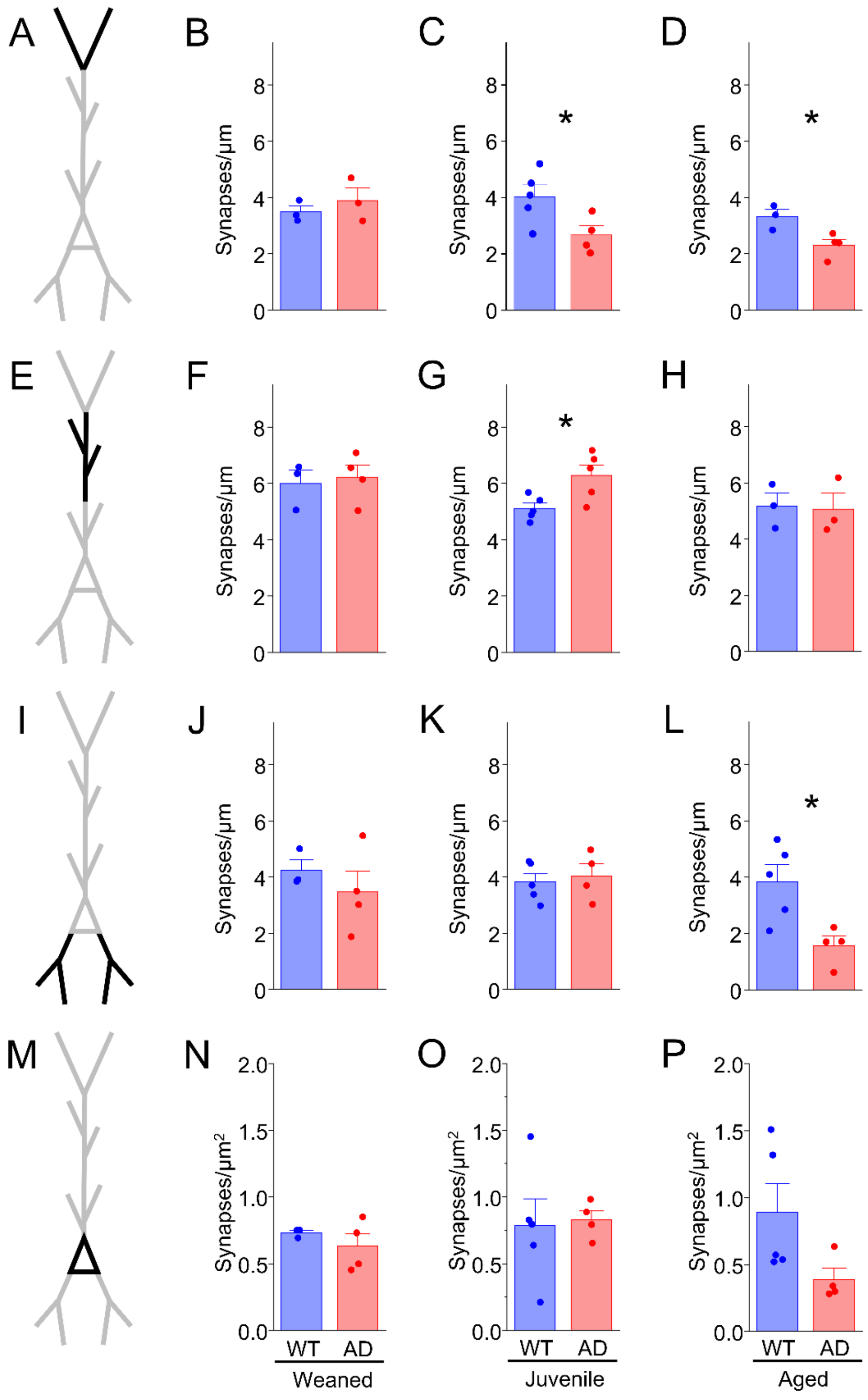

Figure S3. Animal-averaged synapse density measurements in CA1 Pyr compartments in APP/PS1 mice, *related to Figure 3, 5, & 6*.

Synaptic density analysis in cellular compartments averaged by animals from Figures 3(juvenile), 5 (weaned), and 6 (aged).

(A) Schematic of a CA1 Pyr with apical tuft dendrites (black) for analysis in (B-D).
(B) Synapse density is unaltered at tuft dendrites in the weaned AD group (red bar; 3 mice) compared to WT (blue bar; 3 mice). Unpaired $t(4) = -0.823$; $p = 0.4565$.
(C) Synapse density decreases at tuft dendrites in the juvenile AD group (4 mice) compared to WT (5 mice). Unpaired $t(7) = 2.441$; $p = 0.0447$.
(D) Synapse density decreases at tuft dendrites in the aged AD group (4 mice) compared to WT (3 mice). Unpaired $t(5) = 3.049$; $p = 0.0285$.
(E) Same as in (A) but for apical dendrites in *SR-D* (black).
(F) Synapse density is unaltered in apical dendrites from the weaned AD group (3 mice) compared to WT (4 mice). Unpaired $t(5) = -0.321$; $p = 0.7611$.
(G) Synapse density shows a non-significant increase at apical dendrites in the juvenile AD group (5 mice) compared to WT (5 mice). Unpaired $t(8) = -2.765$; $p = 0.0245$.
(H) Synapse density is unaltered in apical dendrites from the aged AD group (3 mice) compared to WT (3 mice). Unpaired $t(4) = -0.149$; $p = 0.8888$.
(I) Same as in (A) except for basal dendrites in *SO* (black).
(J) Synapse density is unaltered on basal dendrites in the weaned AD group (4 mice) compared to WT (3 mice). Unpaired $t(5) = 0.835$; $p = 0.4420$.
(K) Synapse density is unaltered on basal dendrites in the juvenile AD group (4 mice) compared to WT (5 mice). Unpaired $t(7) = -0.430$; $p = 0.6800$.
(L) Synapse density shows a significant decrease on basal dendrites in the aged AD group (4 mice) compared to WT (5 mice). Unpaired $t(7) = 3.042$; $p = 0.0188$.
(M) Same as in (A) but for Pyr soma in *SP* (black).
(N) No change detected in somatic synapse numbers in the weaned AD group (4 mice) compared to WT (3 mice). Unpaired $t(5) = 0.869$; $p = 0.4248$.
(O) No change detected in somatic synapse numbers in the juvenile AD group (4 mice) compared to WT (5 mice). Unpaired $t(7) = -0.187$; $p = 0.8570$.
(P) Somatic synapse density shows a non-significant decrease in the aged AD group (4 mice) compared to WT (5 mice). Unpaired $t(7) = 1.972$; $p = 0.0892$.
All bars represent mean + SEM; asterisks represent $p < 0.05$.

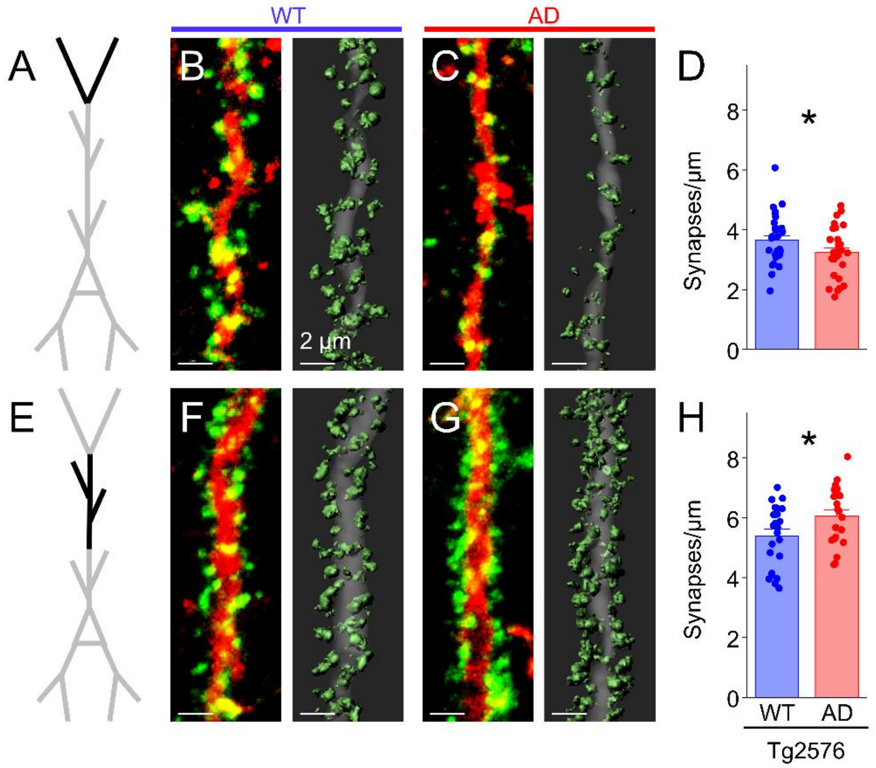

Figure S4. Juvenile Tg2576 mice show similar dendrite-specific synapse gain and loss, *related to Figure 3***.**

Synaptic density analysis in dendritic compartments from CA1 Pyr in Tg2576 mice at 6 weeks of age.

(A) Schematic of a CA1 Pyr highlighting apical tuft dendrites (black) in *SLM* analyzed in (B-D).

(B) Left: Fluorescent image showing FAPpost-marked synaptic puncta (green) and cell fill (red) on a tuft dendritic branch from a WT littermate in the Tg2576 strain. Right: 3D-reconstruction of the fluorescence signal with synapses (light green) and dendrite (grey).

(C) Same as in (B) but from a heterozygous (AD) Tg2576 mouse.

(D) Synaptic density decreases in tuft dendrites in the AD group (32 dendrites from 4 mice) compared to the WT group (blue, 32 dendrites from 4 mice). Unpaired $t(62) = 2.0620$; $p = 0.0434$.

(E-G) Same as in (A-C) but for apical dendrites in *SR-D* (black).

(H) Synaptic density increases at apical dendrites in the AD group (24 dendrites from 4 mice) compared to WT group (24 dendrites from 4 mice). Unpaired $t(46) = -2.338$; $p = 0.0238$.

All bars represent mean + SEM; asterisks represent $p < 0.05$.

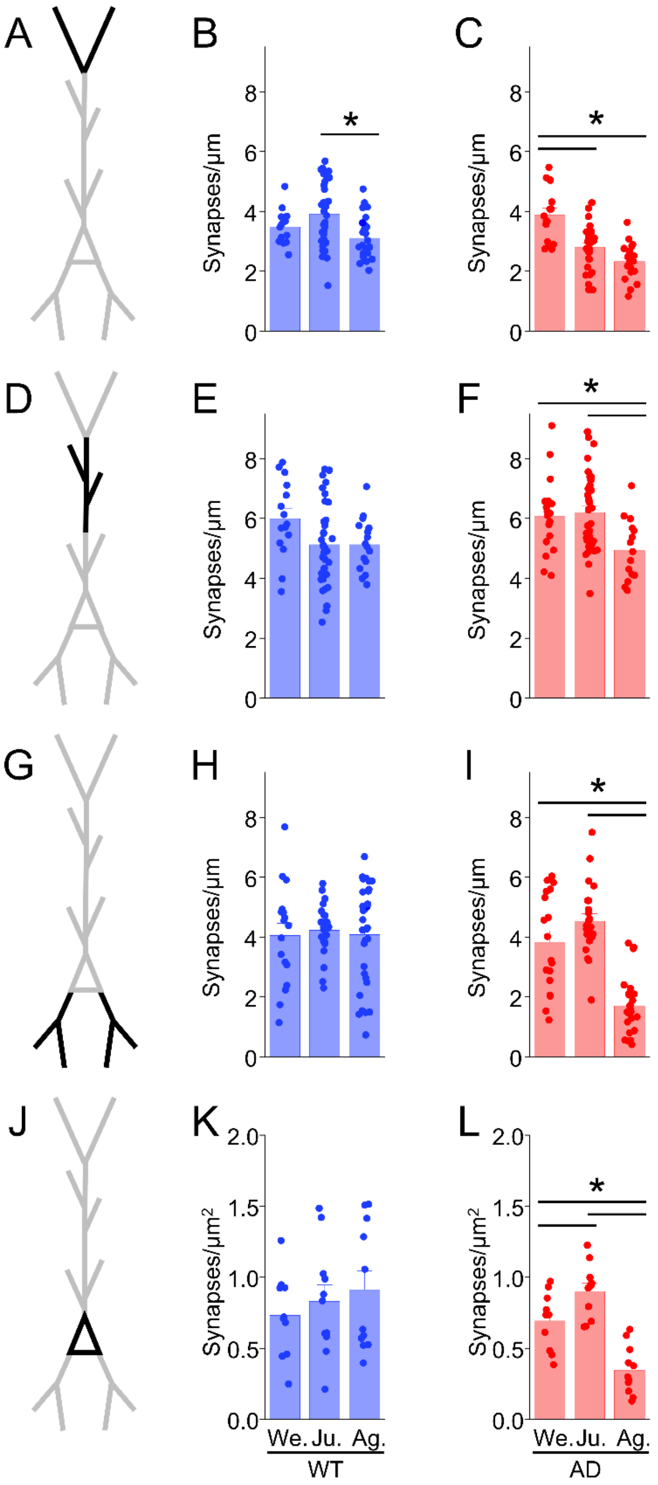

Figure S5. Changes in synapse density in CA1 Pyr compartments across age, *Related to Figure 6 & 7*.

Synaptic density analysis in cellular compartments from Figures 1 (juvenile), 5 (weaned), and 6 (aged) were separated by genotype and compared across ages.

(A) Schematic of a CA1 Pyr with apical tuft dendrites (black) for analysis in (B-C).

(B) Synapse density is significantly altered with age at tuft dendrites in WT mice. One-way ANOVA $F(2,72) = 6.450$; $p = 0.0026$.
(C) Synapse density is significantly altered with age at tuft dendrites in AD mice. One-way ANOVA $F(2,62) = 20.429$; $p = 1.531 \times 10^{-7}$.
(D) Same as in (A) but for apical dendrites in *SR-D* (black).
(E) Synapse density does not change with age in apical dendrites in WT mice. One-way ANOVA $F(2,65) = 2.839$; $p = 0.0658$.
(F) Synapse density is significantly altered with age in apical dendrites in AD mice. One-way ANOVA $F(2,65) = 5.702$; $p = 0.0052$.
(G) Same as in (A) except for basal dendrites in *SO* (black).
(H) Synapse density does not change with age in basal dendrites in WT mice. One-way ANOVA $F(2,70) = 0.096$; $p = 0.9082$.
(I) Synapse density is significantly altered with age in basal dendrites in AD mice. One-way ANOVA $F(2,62) = 32.402$; $p = 2.328 \times 10^{-10}$.
(J) Same as in (A) but for Pyr soma in *SP* (black).
(J) No change detected in somatic synapse numbers with age in WT mice. One-way ANOVA $F(2,29) = 0.562$; $p = 0.5762$.
(L) Somatic synapse density significantly altered with age in AD mice. One-way ANOVA $F(2,29) = 24.636$; $p = 5.591 \times 10^{-7}$.
All bars represent mean + SEM; horizontal bar denotes the pairs that are significantly different in a Tukey's multiple-comparisons corrected test with asterisks representing $p < 0.05$.

SUPPLEMENTARY REFERENCES

S1. Kuljis, D.A., Park, E., Telmer, C.A., Lee, J., Ackerman, D.S., Bruchez, M.P., and Barth, A.L. (2019). Fluorescence-Based Quantitative Synapse Analysis for Cell Type-Specific Connectomics. eNeuro *6*. 10.1523/ENEURO.0193-19.2019.
